# Investigation of Spatial Clustering of Biliary Tract Cancer Incidence in Osaka, Japan: Neighborhood Effect of a Printing Factory

**DOI:** 10.2188/jea.JE20150116

**Published:** 2016-09-05

**Authors:** Yuri Ito, Tomoki Nakaya, Akiko Ioka, Tomio Nakayama, Hideaki Tsukuma, Shinichiro Uehara, Kyoko Kogawa Sato, Ginji Endo, Tomoshige Hayashi

**Affiliations:** 1 Center for Cancer Control and Statistics, Osaka Medical Center for Cancer and Cardiovascular Diseases, Osaka, Japan; 1 大阪府立成人病センター がん予防情報センター; 2 Research Center for Disaster Mitigation of Urban Cultural Heritage, Ritsumeikan University, Kyoto, Japan; 2 立命館大学 歴史都市防災研究所; 3 Department of Preventive Medicine and Environmental Health, Osaka City University Graduate School of Medicine, Osaka, Japan; 3 大阪市立大学 大学院医学研究科 産業医学

**Keywords:** standardized incidence ratio, biliary tract cancer, spatial clusters, cancer registry, 標準化罹患比, 胆管がん, 空間的集積性, がん登録

## Abstract

**Background:**

In 2013, an unusually high incidence of biliary tract cancer among current or former workers of the offset color proof printing department of a printing company in Osaka, Japan, was reported. The purpose of this study was to examine whether distance from the printing factory was associated with incidence of biliary tract cancer and whether incident biliary tract cancer cases clustered around the printing factory in Osaka using population-based cancer registry data.

**Methods:**

We estimated the age-standardized incidence ratio of biliary tract cancer according to distance from this printing factory. We also searched for clusters of biliary tract cancer incidence using spatial scan statistics.

**Results:**

We did not observe statistically significantly high or low standardized incidence ratios for residents in each area categorized by distance from the printing factory for the entire sample or for either sex. The scan statistics did not show any statistically significant clustering of biliary tract cancer incidence anywhere in Osaka prefecture in 2004–2007.

**Conclusions:**

There was no statistically significant clustering of biliary tract cancer incidence around the printing factory or in any other areas in Osaka, Japan, between 2004 and 2007. To date, even if some substances have diffused outside this source factory, they do not appear to have influenced the incidence of biliary tract cancer in neighboring residents.

## Abstract (ja)

**背景:**

2013年、大阪の某印刷会社のオフセット校正印刷部門の現職または元従業員において著しく高い胆管がんの罹患率が報告された。本研究は大阪府がん登録資料を用いて、当該印刷所から居住地までの距離と胆管がん罹患との間に関連があるか、また、当該印刷所付近に胆管がん罹患の集積性が認められるかどうかについて検討することを目的とした。

**方法:**

印刷所との距離に応じて、胆管がんの標準化罹患比を推定した。空間スキャン統計量を用いて、胆管がん罹患の空間的集積の検出を試みた。

**結果:**

男女計、男性、女性ともに、印刷所との距離で区分されたどの地域における住民においても、統計的に有意に高いまたは低い標準化罹患比は観測されなかった。2004～2007年の大阪府を対象にしたスキャン統計量の結果からは、胆管がん罹患が有意に集積している地域はどこにも検出されなかった。

**結論:**

2004～2007年の期間では、印刷所周辺および大阪府内のいずれの地域においても、有意な胆管がん罹患の集積は見られなかった。この印刷所から幾らかの化学物質が漏れ出していたとしても、研究対象期間において近隣住民の胆管がん発生に影響するものではなかったと考えられる。

## INTRODUCTION

In 2013, an unusually high incidence of biliary tract cancer among young current or former workers at the offset color proof department of a printing company in Osaka, Japan, was reported.^1^ In 2014, clinical findings of 17 patients who had worked at this printing factory and had a diagnosis of biliary tract cancer between 1996 and 2012 were reported.^2^^–^^4^ Standardized incidence ratios (SIRs) of biliary tract cancer for workers in this offset color proof printing factory, using the national incidence level as a reference, have been reported to be more than 1000.^5^ Exposure to 1,2-dichloropropane or both 1,2-dichloropropane and dichloromethane at printing factories has been reported to be associated with risk of biliary tract cancer.^2^^,^^6^ If some substances have diffused outside this factory, it is important to establish whether this contamination has influenced the incidence of biliary tract cancer in neighboring residents.

Therefore, we examined whether distance from the printing factory was associated with the incidence of biliary tract cancer and whether there was any clustering of biliary tract cancer incidence around this printing factory in Osaka using data from a population-based cancer registry. A cluster was defined as an area where an unusually high incidence of disease occurs compared with the incidence in the whole study region.

## METHODS

### Data sources

To examine the association of distance from the printing factory with biliary tract cancer incidence, we used incidence data^7^ and population data^8^ by small administrative unit, and compared data with the standard incidence rate^7^ in Osaka, Japan. Osaka Prefecture is located at the approximate center of Japan and has the third largest population (8.8 million) of any prefecture in Japan (eFigure 1A). Osaka Prefecture has 43 municipalities, comprising 33 cities, nine towns, and one village. Osaka City has 24 wards (eFigure 1B). The printing factory is located in Osaka City (eFigure 1B).

Incidence data of biliary tract cancer among residents in Osaka prefecture from 2004 to 2007 was obtained from the Osaka Cancer Registry.^7^ Incidence of biliary tract cancer was identified using codes C22.1 and C24.0 of the International Classification of Diseases, 10th revision. The number of observed incident cases was calculated by sex and patient address using the small geographic area called “Cho-Aza level” in Japan. The Cho-Aza level, which is much smaller than a municipality and a prefecture, is one of the elements of the address system in Japan (eFigure 1B). Of 17 patients who had worked at this factory and had a diagnosis of biliary tract cancer, three incident cases diagnosed with biliary tract cancer between 2004 and 2007 were excluded from the analysis to distinguish occupational exposure from environmental exposure. This study was approved by the data usage committee of the Osaka Cancer Registry of the Osaka Medical Center for Cancer and Cardiovascular Diseases in September 2012 (approval ID: No. 12-0007).

Population data by sex, 5-year age group, and Cho-Aza level in Osaka were obtained from the 2005 National Census to calculate the expected number of incident cases of biliary tract cancer for the Cho-Aza level.^8^

To control for differences in age distribution among areas, we used SIRs. The SIRs of biliary tract cancer by sex and 5-year age group were calculated using whole cases and age-specific population in Osaka in 2004–2007 (see eTable 1). We limited the age range to 0 to 84 years for all analyses because data for the over-85-year age group showed unstable statistical results in the small area that we used in this study.

### Statistical analysis

#### Relationship between distance from the printing factory and biliary tract cancer incidence

We calculated SIRs using the indirect method to control for differences in age distribution among areas categorized by distance from the printing factory (<1.0 km, 1.0 to <2.0 km, 2.0 to <3.0 km, 3.0 to <4.0 km, 4.0 to <5.0 km, and ≥5.0 km). The expected number of cases in each categorized area was obtained from the sum of the products of population in each area and standard incidence rate of biliary tract cancer by 5-year age group and sex in Osaka in 2004–2007. The 95% confidence intervals of SIR were calculated using Fisher’s exact method by assuming a Poisson process.^9^ Distance from the printing factory was used as a categorical variable because the number of incident cases was not sufficient to obtain stable results using distance as a continuous variable. The analysis was performed using the statistical package Stata ver. 13.1. (StataCorp, College Station, TX, USA).^10^

#### Clustering of biliary tract cancer incidence

We also searched for clusters of biliary tract cancer incidence in Osaka using spatial scan statistics (Poisson model). We used SaTScan v.9.1.1 (Martin Kulldorff, Boston, MA, USA), which can detect clusters of infectious and chronic diseases, vectors, and risk of diseases.^11^ A disease cluster is defined as an area where an unusually high incidence of disease occurs compared with the incidence in the whole of the study region. Recently, Kulldorff’s SaTScan^11^ has become the most widely used tool for detecting disease clusters in epidemiological studies, especially for cancer control studies.^12^^–^^18^ A summary of the spatial scan statistics (Poisson model) to identify clusters is shown in eAppendix 1.^12^^,^^17^^,^^19^^,^^20^

To detect significant clusters, a large number of circular windows with varied radii were generated. Each circle window had a different radius and scanned all points of the area. We used the Cho-Aza level as the smallest geographical area to detect clusters. The spatial scan statistics, which were calculated as likelihood ratios, were calculated for all circles. This method, which focuses on the maximum likelihood ratio, can avoid the need for multiple testing to identify clusters. To obtain the *P*-value for the statistically significant test and to define the cluster, we generated 999 replications of a Monte Carlo simulation.

We set the maximum spatial cluster size as 2.0 km to confirm the environmental exposure of chemical substances because, even in the case of asbestos exposure, which can be diffused over a longer distance than chemical substances, the maximum environmental exposure was reported as 2.2 km.^21^ To identify the cluster, we allowed overlapping with the most likely cluster.

## RESULTS

The 2492 cases of biliary tract cancer in Osaka prefecture, Japan, in 2004–2007 included 1528 men and 964 women. The crude incidence rate per 100 000 person-years was 7.24 (9.07 in men and 5.48 in women). The age-standardized incidence rate per 100 000 person-years was 4.37 (5.94 in men and 3.06 in women). Higher incidence rates were observed in the older age group (eTable 1). More detailed descriptive epidemiology of biliary tract cancer in Osaka prefecture has been reported elsewhere.^22^

### Relationship between distance from the printing factory and biliary tract cancer incidence

**Figure 1.**
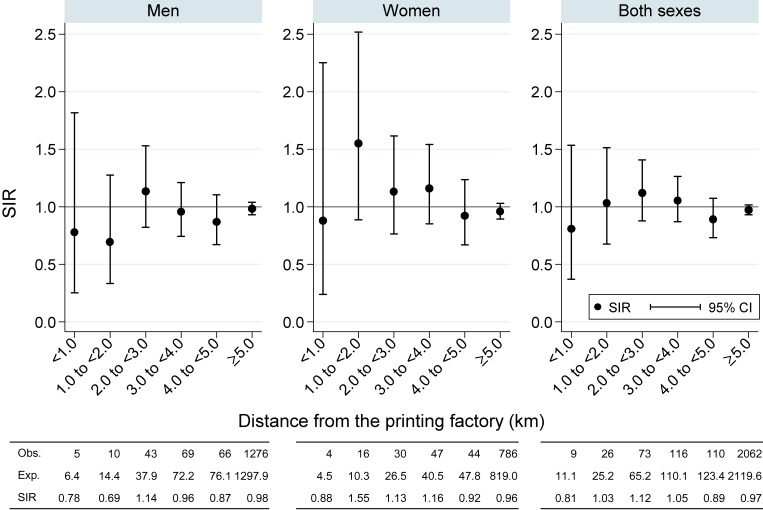
Standardized incidence ratios of residents according to the distance from the printing factory in 2004–2007 in Osaka, Japan.  CI, confidence interval; Exp, expected number of biliary tract cancer cases; Obs, observed number of biliary tract cancer cases; SIR, standardized incidence rate.

The aim of the first set of the analysis was to determine whether the distance from the printing factory was associated with biliary tract cancer incidence. As shown in Figure 1, we did not observe statistically significantly high or low SIRs of residents in any areas categorized by distance from the printing factory.

### Clustering of biliary tract cancer incidence

We have listed the properties of the clusters in increasing order of *P*-value reported by SaTScan for both sexes and by sex in Table 1. According to the scan statistics analysis for both sexes, the most likely cluster was located 4.7 km from the printing factory, but the *P*-value was 0.217 and not statistically significant. The most likely cluster for men was located 8.2 km from the printing factory, and the most likely cluster for women was located 11.7 km from the printing factory; neither cluster was statistically significant. In addition, the radiuses of those clusters were small, and the printing factory was not located inside the clusters. The scan statistics did not show any statistically significant clustering of biliary tract cancer incidence anywhere in Osaka prefecture in 2004–2007.

**Table 1.** Properties of clusters in order of smallest *P*-values reported by SaTScan

Sex	Cluster	Distance from the factory (km)	Radius of the cluster (km)	Number of Cho-Aza^a^ included in a cluster	Likelihood ratio	*P*-value	Observed cases	Expected cases	Standardized incidence ratio
Male	1 (The most likely cluster)	8.2	0.0	1	8.599	0.196	2	0.01	199.1
	2	18.3	1.3	9	6.538	0.816	7	1.20	5.8
	3	8.6	1.2	21	6.441	0.855	23	9.83	2.3
	4	17.6	0.7	7	6.404	0.867	13	3.98	3.3
	5	27.5	0.6	3	5.781	0.973	6	1.00	6.0
	6	11.6	1.2	26	5.352	0.996	27	13.43	2.0

Female	1 (The most likely cluster)	11.7	0.7	4	8.933	0.157	10	1.81	5.5
	2	15.3	0.5	2	7.013	0.633	4	0.27	14.6
	3	4.3	1.7	22	6.896	0.661	30	14.09	2.1
	4	11.2	0.5	16	5.978	0.928	6	0.96	6.3
	5	10.0	1.3	25	5.829	0.953	15	5.37	2.8
	6	18.5	0.9	10	5.327	0.984	6	1.09	5.5
	7	23.0	0.6	4	5.240	0.997	4	0.44	9.0
	8	26.0	1.7	14	5.169	0.997	13	4.59	2.8

Both sexes	1 (The most likely cluster)	4.7	1.2	12	8.978	0.217	32	13.66	2.4
	2	8.2	0.0	1	8.167	0.348	2	0.01	160.4
	3	6.4	0.7	5	5.987	0.952	17	6.43	2.7
	4	8.8	1.9	39	5.777	0.965	59	36.76	1.6
	5	11.2	0.9	37	5.608	0.978	17	6.66	2.6
	6	23.0	0.6	4	5.230	0.996	6	1.11	5.4
	7	3.3	0.8	12	5.155	0.996	19	8.23	2.3

^a^Cho-Aza is the small geographic area which we used in the analysis. It is one of the elements of the address system in Japan and is much smaller than a municipality or a prefecture.

## DISCUSSION

Our study demonstrated that distance from the printing factory was not associated with incidence of biliary tract cancer and that there was no statistically significant clustering of biliary tract cancer incidence around the factory or in any other areas of Osaka between 2004 and 2007.

No results showed statistically significant high SIRs, and there were no significant clusters based on the spatial scan statistics. Therefore, we cannot conclude from our analysis that there is an increased risk of biliary tract cancer incidence around the printing factory.

Only one study examining SIRs for men and women working in the printing industry is available.^23^ Vlaanderen et al reported that, in four Nordic countries (Finland, Iceland, Norway, and Sweden), the SIRs of liver cancer (ICD 10 codes: C22.0 and C22.1) and intrahepatic biliary tract cancer (C22.1) of all printers and related workers were significantly higher than in the general population.^23^ However, no one has yet examined cancer clustering in areas where printing factories are located.

The “Expert Panel on Biliary Tract Cancer at printing factories”, organized by the Ministry of Health, Labour and Welfare, concluded that 16 cases of biliary tract cancer in workers at the printing factory were related to occupational exposure to high concentrations of dichloromethane and 1,2-dichloropropane.^24^ A recent report from November 30, 2014, confirmed that seven workers who were engaged in ink removal operations at small printing factories in Miyagi, Fukuoka, and Hokkaido, Japan, have developed biliary tract cancer from being exposed to highly concentrated 1,2-dichloropropane during their work.^25^ Although the accurate causal relationship between the concentration of exposure and the incidence of biliary tract cancer is still unknown, high-level exposure to these substances in a small space might cause biliary tract cancer.

There are some limitations to our analysis. The population-based cancer registry data did not include data from the occupational database and could not link to these data either. Although we excluded subjects who worked in the printing factory from our analysis, other potential cases related to print workers who worked in other printing factories were not excluded. We were therefore not able to comprehensively account for environmental risks of neighborhood residents in our analysis to examine whether distance from the printing factory was associated with incidence of biliary tract cancer and whether there was clustering of biliary tract cancer incidence around this printing factory in Osaka. In addition, known risk factors of biliary tract cancer, such as infection with hepatitis B and C viruses and liver flukes,^26^ could not be considered in our analysis because of lack of prevalence data on those factors in such a small area of Osaka, Japan.

In conclusion, our study confirmed that the risk of biliary tract cancer was not associated with distance from one specific printing factory in Osaka, Japan. We did not observe spatial clusters of biliary tract cancer incidence in Osaka prefecture during the study period 2004–2007. Although we could not determine whether any substances had diffused outside this factory, we need to examine the future long-term effects of potential environmental contamination on the incidence of biliary tract cancer in residents of areas neighboring the printing factory. Further investigations are also needed in other areas of Japan.

## ONLINE ONLY MATERIALS

eTable 1. Age-specific, crude, and age-standardized incidence rates in Osaka, Japan in 2004–2007.

eFigure 1A. Location of Osaka prefecture in Japan.

eFigure 1B. Municipalities and Cho-Aza in Osaka prefecture.

eAppendix 1. Spatial scan statistics to identify clusters.

Abstract in Japanese.
