# Supplementary material for: Investigation of Spatial Clustering of Biliary Tract Cancer Incidence in Osaka, Japan: Neighborhood Effect of a Printing Factory
Source: J Epidemiol. 2016 Sep 5;26(9):459–63. doi: 10.2188/jea.JE20150116 (PMC5008965; doi:10.2188/jea.JE20150116)
Supplement: eTable 1. [file je-26-459-s001.pdf]

eTable 1. Age-specific, crude, and age-standardized incidence rates in Osaka, Japan, in 2004-2007

Age, years	Men			Women			Both sexes		
	Cases[a]	Person-years[b]	Incidence rate[c]	Cases[a]	Person-years[b]	Incidence rate[c]	Cases[a]	Person-years[b]	Incidence rate[c]
Age-specific incidence rate									
0-4	0	802,609	0	0	765,350	0	0	1,567,959	0
5-9	0	846,800	0	0	806,005	0	0	1,652,805	0
10-14	0	823,510	0	0	784,634	0	0	1,608,144	0
15-19	0	894,096	0	0	860,156	0	0	1,754,252	0
20-24	0	1,064,434	0	3	1,050,847	0.29	3	2,115,281	0.14
25-29	1	1,158,403	0.09	0	1,196,770	0	1	2,355,173	0.04
30-34	3	1,395,279	0.22	1	1,431,856	0.07	4	2,827,135	0.14
35-39	7	1,302,033	0.54	5	1,328,238	0.38	12	2,630,271	0.46
40-44	8	1,126,703	0.71	6	1,147,532	0.52	14	2,274,235	0.62
45-49	16	979,897	1.63	14	1,002,395	1.40	30	1,982,292	1.51
50-54	58	1,086,714	5.34	27	1,132,024	2.39	85	2,218,738	3.83
55-59	141	1,361,386	10.36	66	1,431,024	4.61	207	2,792,410	7.41
60-64	185	1,275,649	14.50	98	1,354,108	7.24	283	2,629,757	10.76
65-69	258	1,072,589	24.05	138	1,146,638	12.04	396	2,219,227	17.84
70-74	365	828,449	44.06	171	948,170	18.03	536	1,776,619	30.17
75-79	280	541,813	51.68	221	715,422	30.89	501	1,257,235	39.85
80-84	206	278,453	73.98	214	493,520	43.36	420	771,973	54.41
Crude incidence rate									
0-84	1,528	16,838,817	9.07	964	17,594,689	5.48	2,492	34,433,506	7.24
Age-standardized incidence rate[d]			5.94			3.06			4.37

[a] Cases who were detected in 2004-2007

[b] Person-years in 2004-2007 in each age-specific group

[c] Incidence rate per 100,000 person-years

[d] Standardized using the direct method with the Japanese population in 1985 as the standard population
