# Supplementary figures and images for: Investigation of Spatial Clustering of Biliary Tract Cancer Incidence in Osaka, Japan: Neighborhood Effect of a Printing Factory
Source: J Epidemiol. 2016 Sep 5;26(9):459–63. doi: 10.2188/jea.JE20150116 (PMC5008965; doi:10.2188/jea.JE20150116)

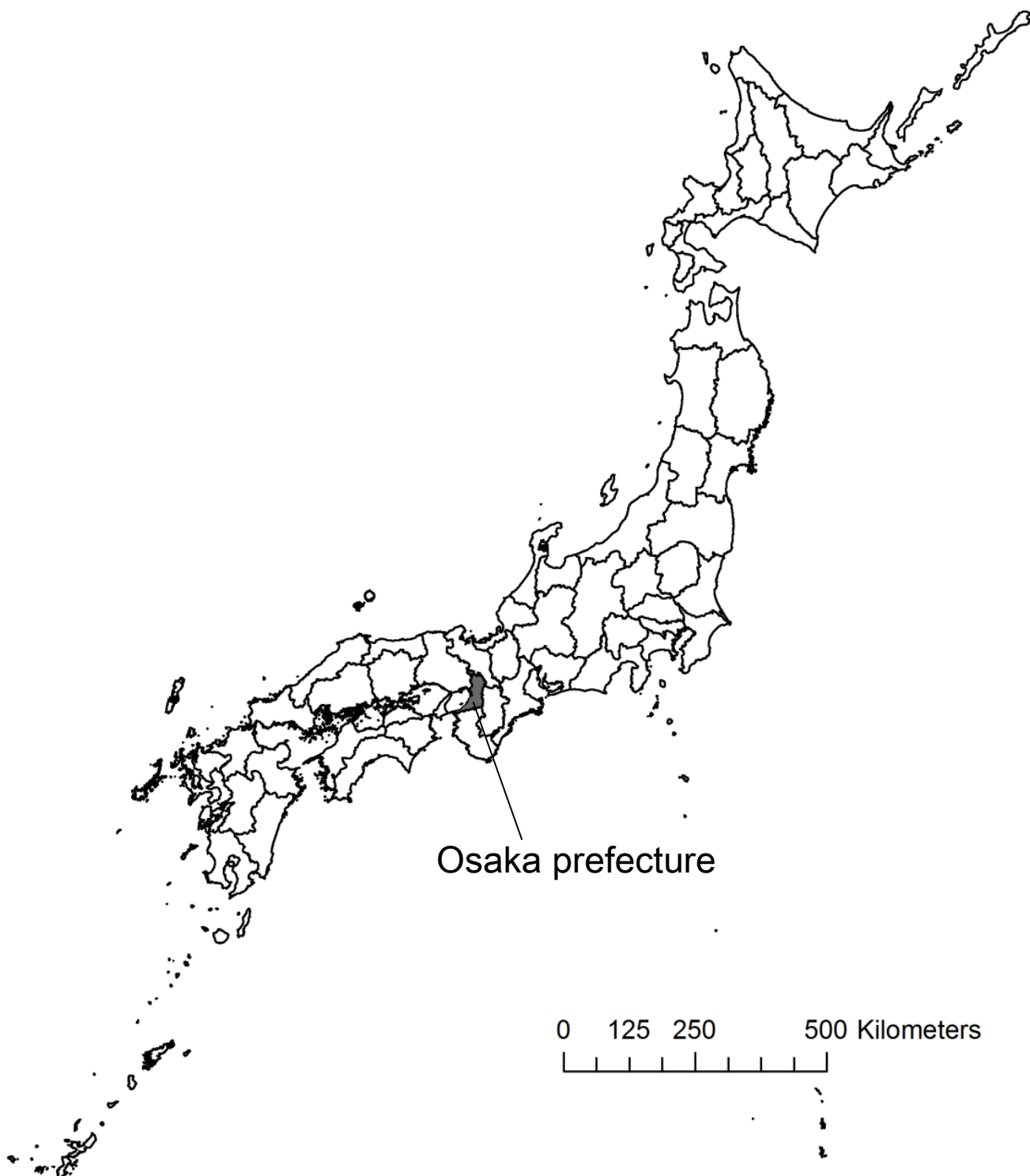

eFigure 1A. Location of Osaka prefecture in Japan

Supplement: eFigure 1A. [file je-26-459-s002.pdf]
