# Supplementary material for: Investigation of Spatial Clustering of Biliary Tract Cancer Incidence in Osaka, Japan: Neighborhood Effect of a Printing Factory
Source: J Epidemiol. 2016 Sep 5;26(9):459–63. doi: 10.2188/jea.JE20150116 (PMC5008965; doi:10.2188/jea.JE20150116)
Supplement: eFigure 1B. [file je-26-459-s003.pdf]

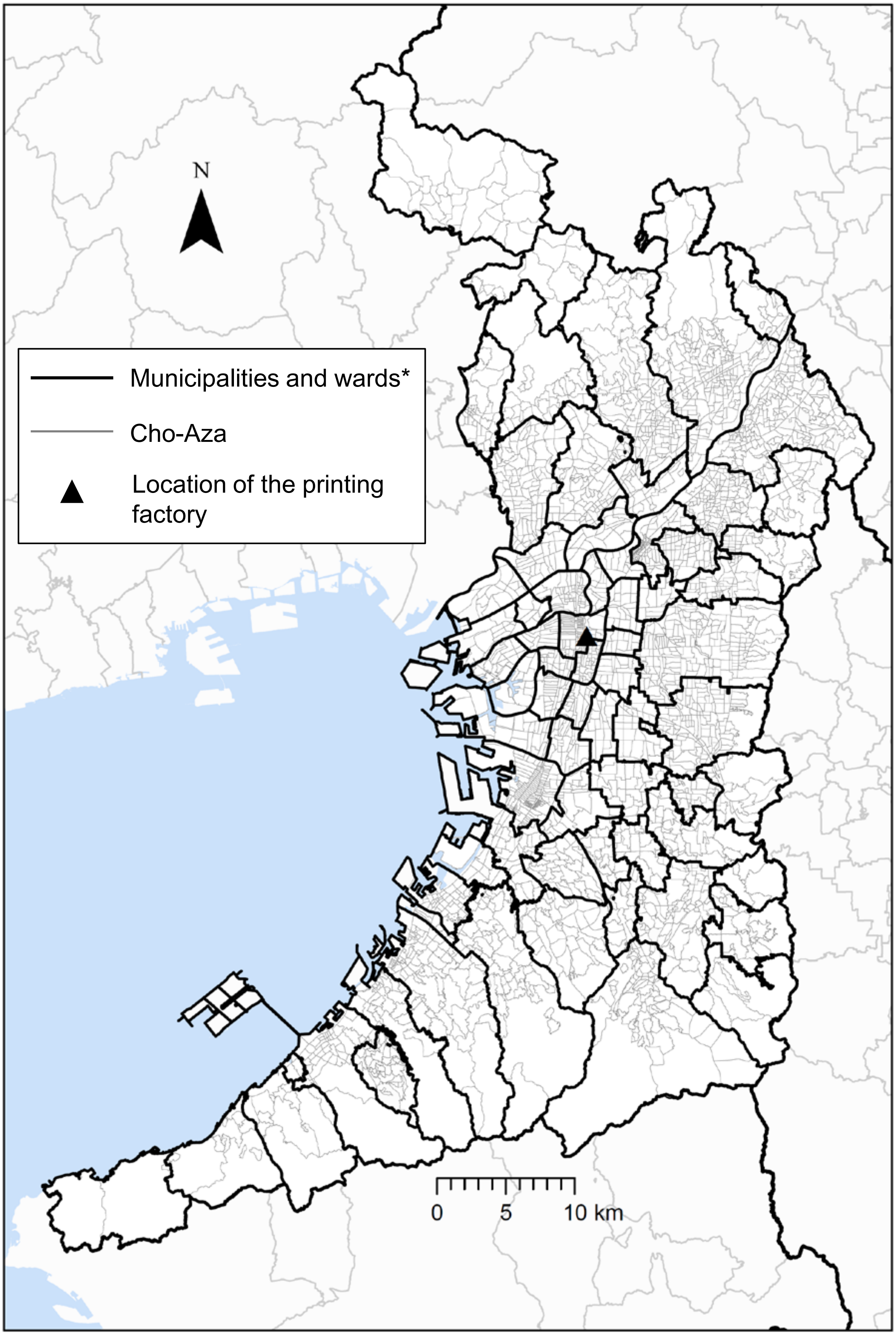

eFigure 1B. Municipalities and Cho-Aza in Osaka prefecture

* Osaka prefecture has 43 municipalities, comprising 33 cities, 9 towns, and 1 village. Osaka City has 24 wards.
