# Supplementary material for: Investigation of Spatial Clustering of Biliary Tract Cancer Incidence in Osaka, Japan: Neighborhood Effect of a Printing Factory
Source: J Epidemiol. 2016 Sep 5;26(9):459–63. doi: 10.2188/jea.JE20150116 (PMC5008965; doi:10.2188/jea.JE20150116)
Supplement: eAppendix 1. [file je-26-459-s004.pdf]

eAppendix 1: spatial scan statistics to identify cluster

Kulldorff's spatial scan statistics set the null hypothesis as disease incidence that occurs randomly in a geographic area, and the expected level of incidence is proportional to the population at risk based on risk factors, such as sex and age. The alternative hypothesis is that an area of high incidence exists compared with outside the area and that the risk is higher than that of chance alone.

To identify a cluster, first, a large number of circular windows with varied radii are generated. The radius of a circle increases from '0' to a user-defined maximum radius. At each point area (Cho-Aza), a spatial scan statistic is calculated within a circle centered in the point area with each different radius.

Second, a likelihood ratio for each circle is calculated to detect a potential cluster. Under the Poisson assumption, the likelihood ratio for a specific window is proportional to:

$$\left(\frac{c}{E[c]}\right)^{c}\left(\frac{C-c}{C-E[c]}\right)^{C-c} I(c > E[c])$$

where C is the total number of cases, c is the observed number of cases within the circle, and $E[c]$ is the expected number of cases within the circle under the null hypothesis. $C - E[c]$ is the expected number of cases outside the circle, $C - c$ is the observed number of cases outside the circle. $\mathrm{I}(c > E[c])$ is an indicator function, which is equal to 1 when $c > E[c]$ and 0 otherwise, when we detect a high incidence area. For each circle at all small area points with varied radii, the likelihood ratio is calculated. By focusing on the maximum likelihood ratio, this method can avoid multiple testing to identify clusters. To obtain the p-value for a statistical significance test, we generate 999 replications of Monte Carlo simulation. The Monte Carlo simulation in the SaTScan used 999 replications as the default setting. This could be changed to any other number ending in 999, such as 1,999, 9,999 or 99,999 as the 999 ending for the replication number produces a 'nice looking' p-value. Monte Carlo hypothesis testing obtains

a p-value by comparing the rank of the maximum likelihood from the real data set with the maximum likelihood from the random data sets. The p-value is then obtained from the rank of the real data set divided by (1 + number of simulation).[1]

The cluster is identified by the circle window with the lowest p-value, which is called the "primary cluster" or the "most likely cluster".

1. SaTScan TM User Guide for version 9.3 [homepage on the Internet]; 2014 [cited 9 Mar 2015]. Available from: http://www.satscan.org/cgi-bin/satscan/register.pl/SaTScan_Users_Guide.pdf?todo=process_userguide_download
