# Supplementary material for: Investigation of Spatial Clustering of Biliary Tract Cancer Incidence in Osaka, Japan: Neighborhood Effect of a Printing Factory
Source: J Epidemiol. 2016 Sep 5;26(9):459–63. doi: 10.2188/jea.JE20150116 (PMC5008965; doi:10.2188/jea.JE20150116)
Supplement: Abstract in Japanese. [file je-26-459-s005.pdf]

大阪府における胆管がん罹患の空間的集積性の検討：印刷所の近隣影響

伊藤ゆり[1]　中谷友樹[2]　井岡亜希子[1]　中山富雄[1]　津熊秀明[1]
上原新一郎[3]　佐藤（粉河）恭子[3]　圓藤吟史[3]　林朝茂[3]

1　大阪府立成人病センター　がん予防情報センター
2　立命館大学　歴史都市防災研究所
3　大阪市立大学　大学院医学研究科　産業医学

背景
　2013年、大阪の某印刷会社のオフセット校正印刷部門の現職または元従業員において著しく高い胆管がんの罹患率が報告された。本研究は大阪府がん登録資料を用いて、当該印刷所から居住地までの距離と胆管がん罹患との間に関連があるか、また、当該印刷所付近に胆管がん罹患の集積性が認められるかどうかについて検討することを目的とした。

方法
　印刷所との距離に応じて、胆管がんの標準化罹患比を推定した。空間スキャン統計量を用いて、胆管がん罹患の空間的集積の検出を試みた。

結果
　男女計、男性、女性ともに、印刷所との距離で区分されたどの地域における住民においても、統計的に有意に高いまたは低い標準化罹患比は観測されなかった。2004～2007年の大阪府を対象にしたスキャン統計量の結果からは、胆管がん罹患が有意に集積している地域はどこにも検出されなかった。

結論
　2004～2007年の期間では、印刷所周辺および大阪府内のいずれの地域においても、有意な胆管がん罹患の集積は見られなかった。この印刷所から幾らかの化学物質が漏れ出していたとしても、研究対象期間において近隣住民の胆管がん発生に影響するものではなかったと考えられる。

キーワード：標準化罹患比、胆管がん、空間的集積性、がん登録
